# Supplementary material for: Rainbow peacock spiders inspire miniature super-iridescent optics
Source: Nat Commun. 2017 Dec 22;8:2278. doi: 10.1038/s41467-017-02451-x (PMC5741626; doi:10.1038/s41467-017-02451-x)
Supplement: Supplementary file 1 — Supplementary Information [file 41467_2017_2451_MOESM1_ESM.pdf]

Supplementary Note 1. Conventional 2D grating equation

Eq. 1 governs the angular locations of the principal intensity maxima when light of wavelength λ is diffracted from a horizontal grating of groove spacing d_h (Supplementary Fig. 7a). The grating equation reveals that only the spectral orders for which $|m_h\lambda_h/d_h| < 2$ can exist and hence determines the specific number of allowed modes for the flat grating. However, due to the complex geometry of the prism and foil gratings (i.e. the superposition of microscopic shape and vertical surface nanogratings), the diffraction profiles are rather complex as shown in Fig. 6h&i. The diffraction profile by the microscopic horizontal grating also follows Eq. 1 and it allows up to 47 orders for d = 10 μm in the same optical region (Supplementary Fig. 7b).

Supplementary Note 2. Dispersion in the context of diffraction grating

Under a very specific context when discussing diffraction gratings, the term dispersion can be defined as "a measurement of the separation (either angular or spatial) between diffracted light of different wavelengths" according to the DIFFRACTION GRATING HANDBOOK[1]. Which can be further separated into angular dispersion – the spectral range per unit angle; and linear dispersion – the spectral range per unit length. Not to be confused with another, and maybe more familiar type of dispersion, the chromatic dispersion, which is defined as the separation of

light due to varies wavelength dependent refractive indices of a material, such as the continuous rainbow spectrum produced by a prism.

Supplementary Note 3. Nano-grating/micro-geometry interaction

In order to decouple the effects of microscopic shape versus the surface nanograting of the foil Foil grating structure, we analysed their respective contribution with FEM simulation of overall geometry with (Fig. 4c) and without (Fig. 4d) the surface nanogratings. The foil-shaped structure without surface nanogratings (i.e. a lenticular prism) results in straight lines in the simulated scattering spectra (Supplementary Fig. 6b) caused by the horizontal periodicity of a unit cell, and such observed locations of these diffraction orders can be directly calculated using the grating equation (Eq. 1).

Supplementary Note 4. The cause of the banded pattern

The banded diffraction profile is produced by the interaction between two individual mechanisms. First, the reverse-ordered diffraction pattern that results from vertical surface nanogratings. Second, the regular ordered diffraction pattern (Supplementary Fig. 6) that results from multiple microscale prism/foil-shaped structures (Fig. 4d) acting as large period

horizontal microgratings (Supplementary Fig. 6). This interference resulted from the fact that these structures were fabricated side-by-side in parallel (Fig. 4f&g).

Supplementary Note 5. Spider visual acuity

M. robinsoni and *M. chrysomelas* are not likely to be able to discern the microscopic rainbow patterns (Fig. 3a) resulting from individual scales because – Assuming the distance between a male and a female during courtship is 1 cm (10,000 μm). We know from SEM/TEM micrographs (Fig. 2) that the width of a single abdominal rainbow-iridescent scale is ~10 μm, which harbours a double rainbow pattern, meaning each of the rainbow pattern spans ~5 μm in width. For a *M. robinsoni* or *M. chrysomelas* female to be able to resolve this rainbow pattern, she needs to have a minimum acuity of α, where $\tan^{-1}(\alpha) = 5/10{,}000$. In other words, the female needs to have an inter-receptor angle smaller than 1.7’ to be capable of seeing the microscopic rainbow patterns, which is beyond the smallest known inter-receptor angle (2.4’) in spiders (i.e., spiders that have the highest acuity – *Portia* spp.)[2].

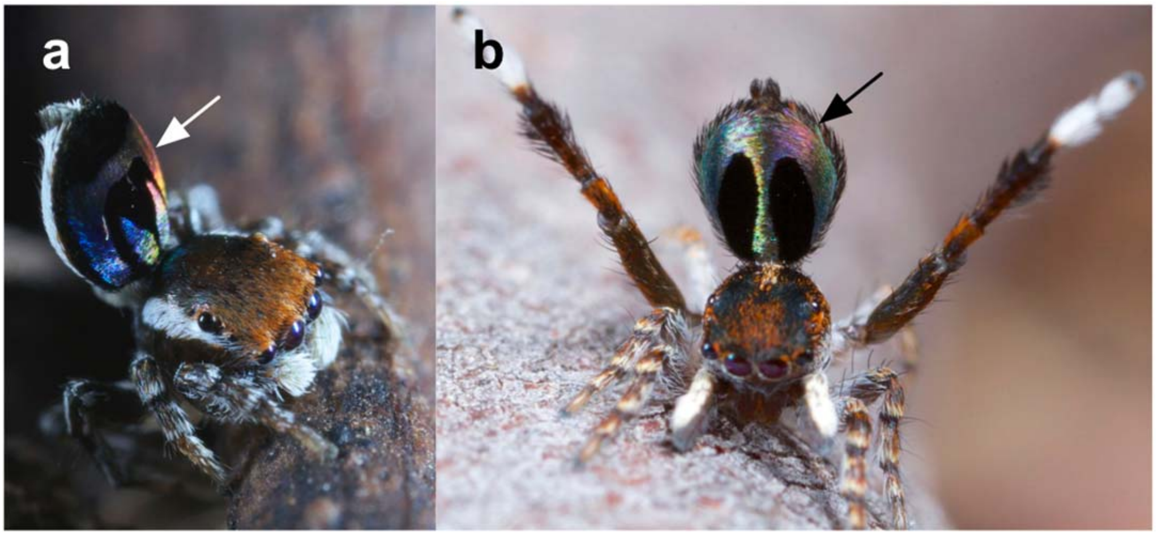

Supplementary Figure 1 | Images of *M. robinsoni* (a) and *M. chrysomelas* (b) engaged in their courtship dancing postures. Arrows show that they flatten and raise their abdomen to showcase the rainbow-iridescence to the potential mates.

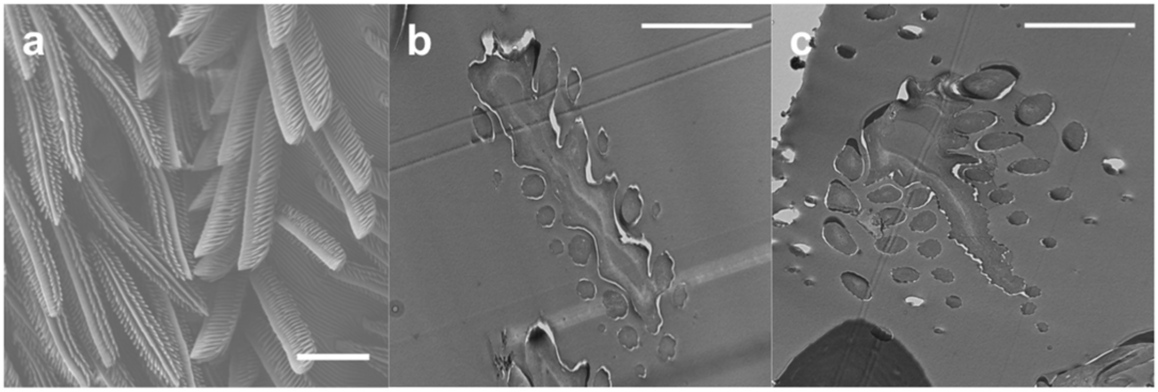

Supplementary Figure 2 | Electron micrographs for the black scales of *M. robinsoni*. a, A SEM micrograph showing randomly orientated brush-like abdominal black scales. Scale bar: 20 μm. **b**&**c**, TEM micrographs showing the coronal (**b**) and transverse (**c**) views of a brush-like abdominal black scale. Granularly and heterogeneously deposited eumelanin is observed inside the black scale. Scale bar: 5 μm

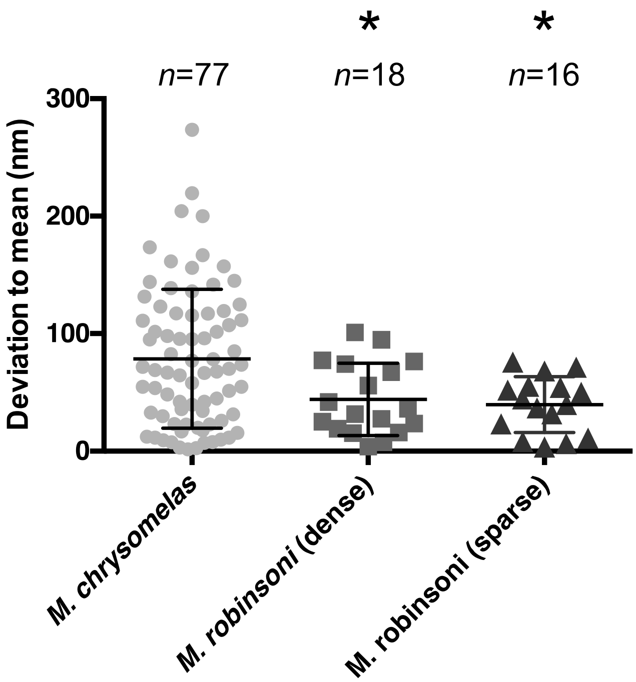

Supplementary Figure 3 | The grating period for the iridescent scales of *M. robinsoni* is more regular than that of *M. chrysomelas*. The distance of each individual spacing to the mean spacing ($D_{individual}$ - D_{mean}) of the gratings are plotted (error bars: mean±*s.d.* Standard deviations are calculated based on all data points (*n*) of each data set.). The averaged "deviation to mean" values of *M. robinsoni* are significantly smaller (* represents $p \leq 0.05$) than that of *M. chrysomelas*, meaning the grating period is more regular in *M. robinsoni* than that in *M. chrysomelas*.

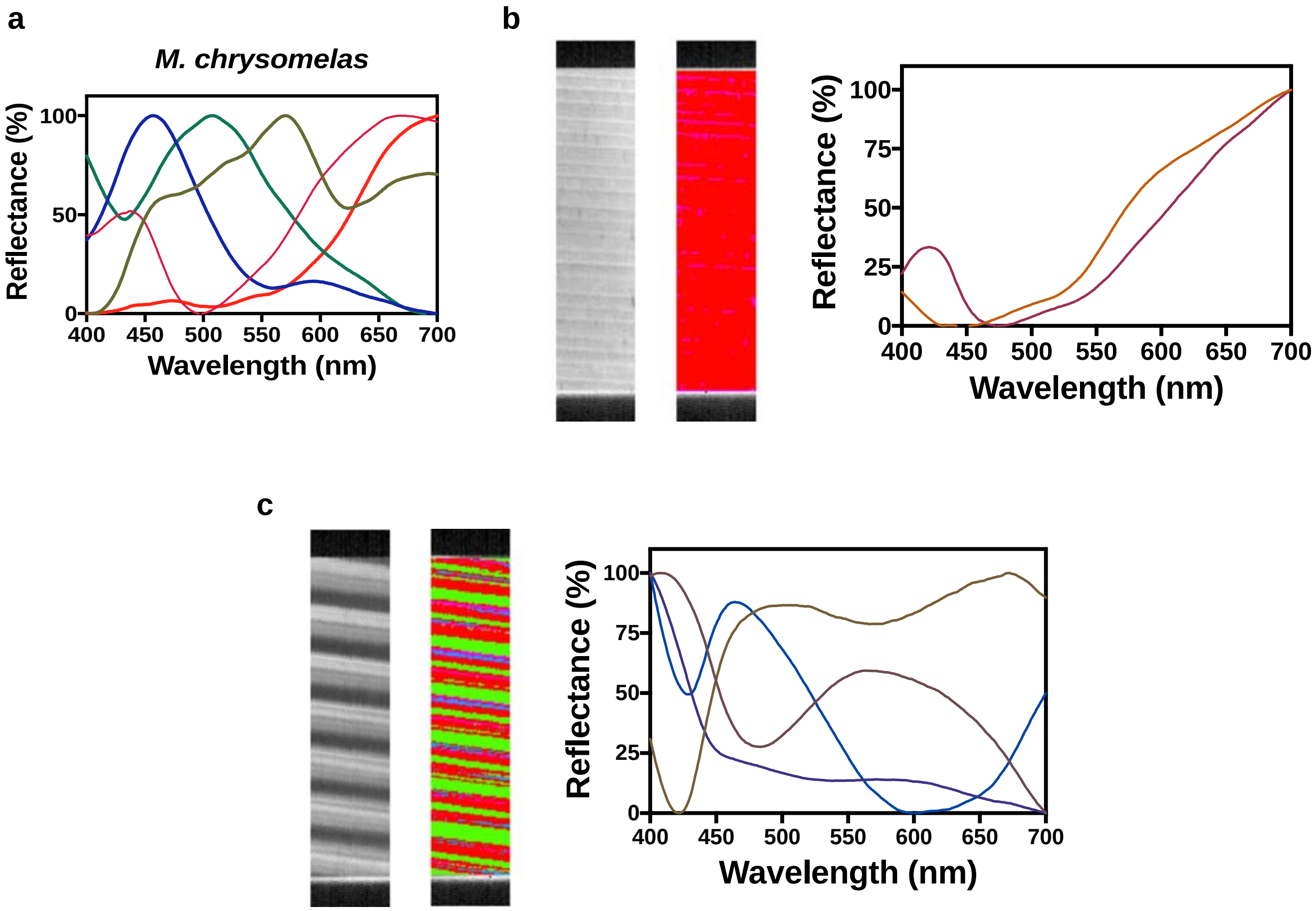

Supplementary Figure 4 | Hyperspectral analysis results. a, The reflectance spectra for the iridescent scales of *M. chrysomelas* collected by the hyperspectral imaging system show an array of rainbow colours. **b-c**, From left to right: the monochrome image, false-colour hyperspectral image, and the reflectance spectra collected by the hyperspectral imaging system from the flat (**b**) and prism (**c**) gratings. **b**, Shows predominantly monochromatic colour. **c**, Shows light diffraction with low resolving power. Hence, the prism grating produces the diffraction pattern with colours of low purity and saturation as seen in Fig. 6d inset. The colours of the reflectance spectra curves are estimated based on the “spec2rgb” function in R script “pavo”[3]. The colours in the false-colour hypersepctral images are arbitrarily assigned, each colour represents a different reflectance spectrum.

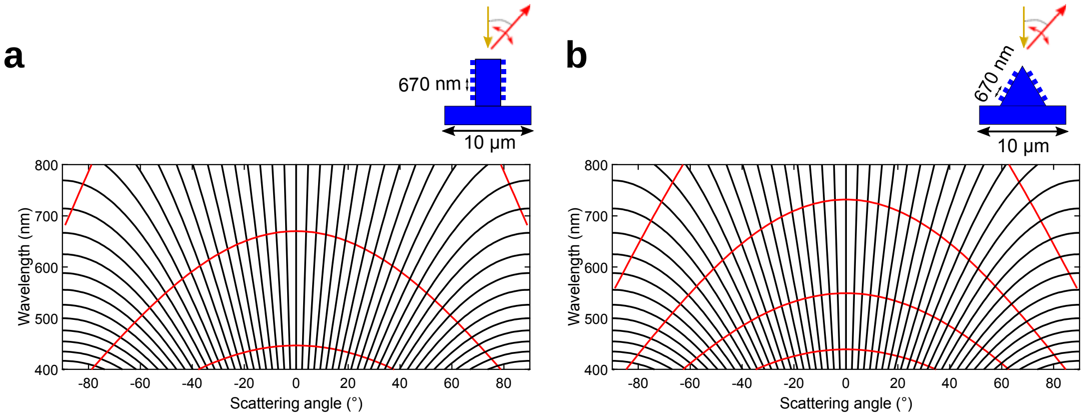

Supplementary Figure 5 | Analytical modelling of complex grating structures. **a**, Superposition of horizontal micro-grating (Eq. 1, black lines) and vertically aligned nano-grating (Eq. 2, red lines). **b**, Superposition of horizontal micro-grating (Eq. 1, black lines) and triangular (Eq. 3, red lines) nano-grating.

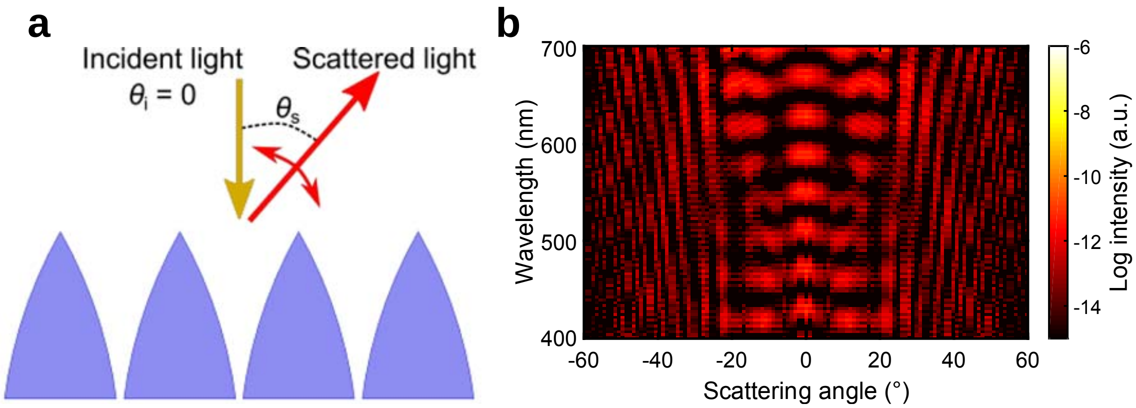

Supplementary Figure 6 | The simulated variable angle scattering spectra for the lenticular prism structures only. a, The simulation schematic for the microscopic lenticular prism structures without the surface nanogratings (Fig. 4d). **b**, The simulated result shows a normal diffraction pattern similar to that of the flat grating (Fig. 6g) with more allowed spectral orders (denser lines) due to the large period (~10 µm).

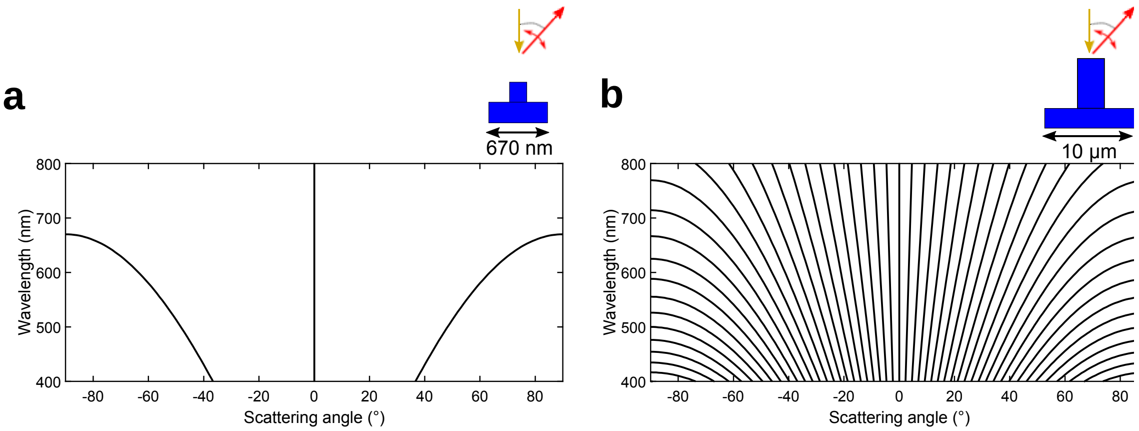

Supplementary Figure 7 | Analytical modelling of horizontal grating structures. **a**, Horizontal nano-grating with 670 nm period. **b**, Horizontal micro-grating with 10 μm period.

Supplementary References

1. Palmer, C. & Loewen, E. DIFFRACTION GRATING HANDBOOK sixth edn. (Newport Corporation, 2005).
2. Land, M. F. in Neurobiology of Arachnids (Barth, F. G.) Ch. IV (Springer Berlin Heidelberg, 1985).
3. Maia, R., Eliason, C. M., Bitton, P.-P., Doucet, S. M. & Shawkey, M. D. pavo: an R package for the analysis, visualization and organization of spectral data. *Methods Ecol. Evol.* **4,** 906–913 (2013).
